# VineColD: an integrative database for global historical tracing and real-time monitoring of grapevine cold hardiness

**DOI:** 10.1093/database/baaf055

**Published:** 2025-09-27

**Authors:** Hongrui Wang, Jason P Londo

**Affiliations:** School of Integrative Plant Science, Horticulture Section, Cornell University-Cornell AgriTech, 635 W North St, Geneva, NY 14456, United States; School of Integrative Plant Science, Horticulture Section, Cornell University-Cornell AgriTech, 635 W North St, Geneva, NY 14456, United States

## Abstract

Cold hardiness is a crucial physiological parameter that determines the survival of grapevines during the dormant season. Accurate modelling and large-scale prediction of grapevine cold hardiness are essential for assessing the potential geographic distribution of grapevine cultivation, quantifying the impact of climate change on grapevine habitats, and ensuring the sustainability of the grape and wine industries worldwide in the regions characterized by cool or cold dormant seasons. However, until now, no comprehensive database has been available. In this research, we combined advanced automated machine learning techniques with extensive historical and current weather data to create an integrative database for grapevine cold hardiness: VineColD (https://cornell-tree-fruit-physiology.shinyapps.io/VineColD/). We developed the NYUS.2.1 model, an automated machine learning-based system for predicting grapevine cold hardiness and applied it to global historical weather data from 17,985 curated weather stations between latitudes 30° and 55° in both hemispheres from 1960 to 2024, resulting in the development of an integrative grapevine cold hardiness database and monitoring system, VineColD. VineColD integrates both a global historical dataset and a daily updated regional cold hardiness system, offering a comprehensive resource to study grape cold hardiness for 54 grapevine cultivars. The platform provides multiple download options, from single-station data to complete datasets, and the interactive multifunctional R Shiny application facilitates data analysis and visualization. VineColD delivers critical insights into the impact of climate change on grapevine cultivation and supports a range of analytical functions, making it a valuable tool for grape growers and researchers.

## Introduction

The distribution of woody perennials across the world is predominantly influenced by climatic factors, with temperature extremes in both summer and winter playing a crucial role in determining their survival [1, 2]. Climate change, characterized by an increased frequency of unusual temperature extremes during the growing season or the dormant season, poses a threat to these species, including economically important perennial crops such as grapevine [3–5]. In the viticulture regions characterized by cool or cold dormant season and established close to the temperature boundaries, extreme low temperatures in winter that exceed grapevine cold hardiness and late spring frosts that damage fragile fruitful tissues after budbreak are the leading causes of crop loss, affecting the ongoing viability and potential expansion of the grape and wine industries in these regions [6–8]. The accurate prediction of dormant season physiology, such as bud cold hardiness and budbreak timing, can be used as guidance for the cultivation of grapevines in changing winters under climate change.

Our current understanding of grapevine dormant season physiology is depicted as a U-shaped pattern of cold hardiness, which exhibits a dynamic balance between cold acclimation (gain of cold hardiness, typically in late fall) and deacclimation (loss of cold hardiness, typically in late winter). The equilibrium between these phases shifts in response to chilling accumulation and daily temperature fluctuations throughout the dormant season [9–19]. While bud cold hardiness determines if grapevines can survive extreme cold events in winter, it is also a crucial physiological trait that influences early spring phenology, including the timing of budbreak [20]. The modelling of grapevine dormant physiology is therefore centred on the prediction of bud cold hardiness. Currently, the benchmark models for grapevine cold hardiness prediction include WAUS.1, WAUS.2, WIUS.2, NYUS.1, NYUS.2, and an unnamed recurrent neural network (RNN)-based model. The WAUS.1 and its successor WAUS.2 were developed in Washington, USA. These models are mechanistic models where continuous changes in bud cold hardiness, estimated as the lethal temperature that kills 50% of primary buds (LT50), are arbitrarily phased with incremental time steps based on daily maximum and minimum temperature and cultivar-specific parameters [9, 21]. WIUS.1 is derived from the modelling method of WAUS.2 but is trained using additional LT50 data from field-collected data for interspecific cold-hardy cultivars grown in Wisconsin, USA [22]. The NYUS.1 model, a recent mechanistic model with empirically derived biological parameters, has been developed to incorporate a phased integration of cold acclimation and deacclimation responses based on the latest insights into the dynamics of dormancy-related cold hardiness in grapevines [23]. The RNN-based model, developed in Washington, USA, is a deep learning model that uses unstructured time series weather data to predict the whole season bud LT50. The model was trained through multitask learning, which leverages data across multiple grape cultivars to improve prediction accuracy, especially for cultivars with limited training data, such as Lemberger and Gewurztraminer [24]. A key advantage of this approach is its simplicity in the modelling process: There is no need to generate cultivar-specific biological parameters or preprocess weather data into engineered features. However, this modelling strategy typically requires (e.g. millions of observations) very large datasets to achieve robust performance.

Beyond the modelling technique and the restricted range of cultivars available for cold hardiness prediction, a notable limitation of all these models is that they were developed using data from a single location, potentially causing them to become excessively tailored to specific local conditions. For example, the underperformance of WAUS.1 and WAUS.2 has been reported when applying these models in other locations with different climate conditions [23, 25, 26]. The final model, NYUS.2, was developed using automated machine learning (Auto-ML) and trained with 10,157 LT50 data points from nine locations and 72 features extracted from air temperature (engineered temperature parameters such as chilling and heat accumulation). This model has demonstrated exceptional robustness and outperformed the previous mechanistic models in the prediction of the cold hardiness of 45 cultivars in various viticultural regions in North America [26]. In addition to leveraging available hardware for enhanced computational speed through the Auto-ML engine, the high site-transferability of this model enables large-scale and multilocation tracing and forecasting of grapevine bud cold hardiness/cold damage, which is essential for the global assessment of the impact of climate change on grapevine dormant season physiology.

In this project, we coupled NYUS.2.1, an updated NYUS.2 model, with GHCNd (Global Historical Climatology Network daily) to calculate grapevine cold hardiness and potential cold damage of 54 grapevine cultivars at accessible weather stations within the latitudes 30°–55° viticultural zone of both hemispheres over 64 dormant seasons from 1960 to 2024. Furthermore, we have created a real-time monitoring system that offers daily updates on grapevine cold hardiness for the major viticultural regions with cool or cold dormant seasons in North America. By combining various sources of grapevine cold hardiness prediction data and actual measurements with previous and current weather records, we developed an integrative database named VineColD (grapeVine Cold hardiness Database, https://cornell-tree-fruit-physiology.shinyapps.io/VineColD/). VineColD is now accessible through a web-based interactive platform that supports diverse functionalities such as data analysis and visualization, as well as uploading and downloading of data, thereby providing a comprehensive tool for grape growers and researchers to navigate and adapt to the challenges posed by climate change.

## Methods and data collection

**Figure 1.**
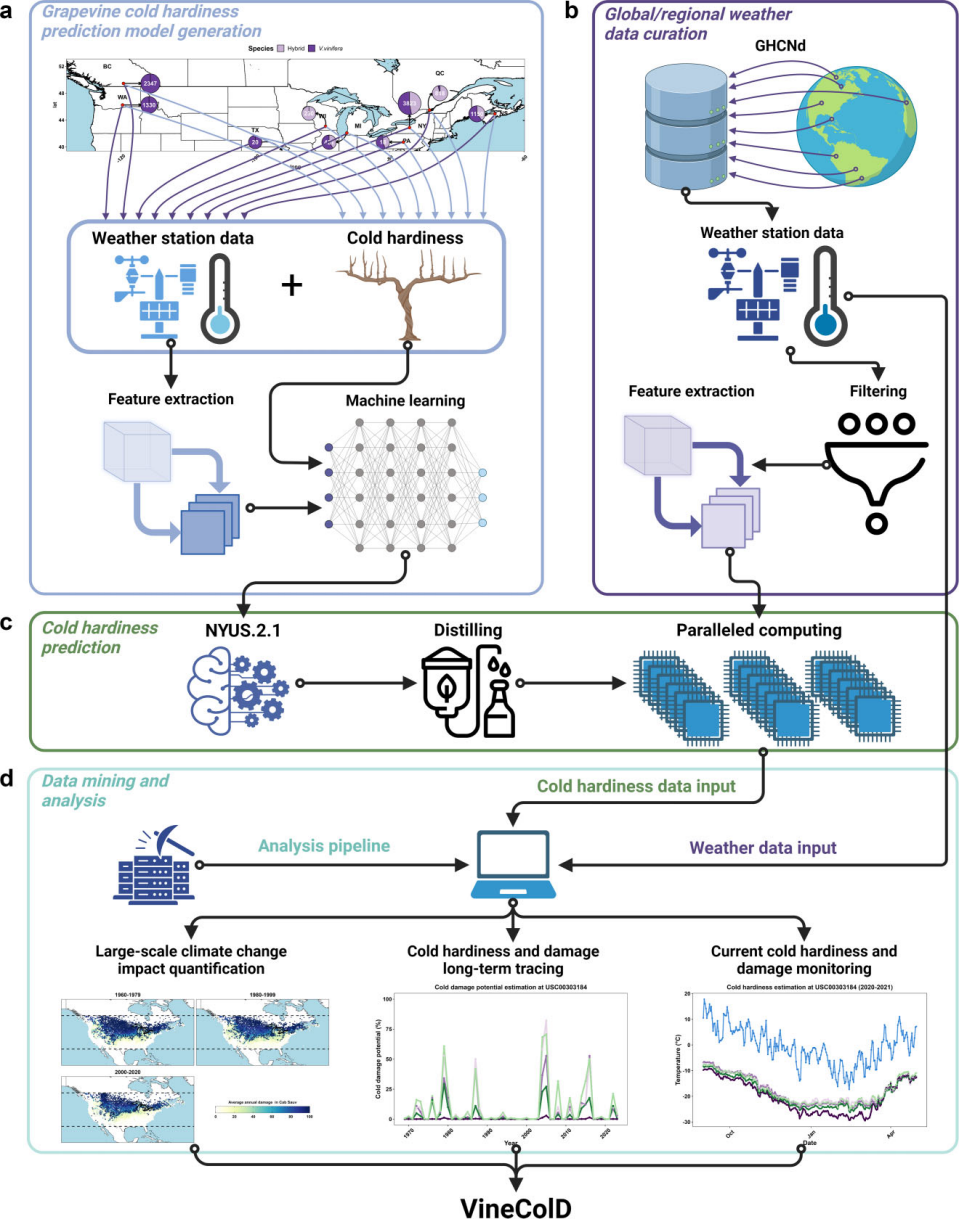
Schematic representation of the development of VineColD. (a) Generation of the new grapevine cold hardiness prediction model, NYUS.2.1; (b) curation of global and regional weather data; (c) computation of grapevine cold hardiness prediction; and (d) data processing and analysis of predicted grapevine cold hardiness for the construction of VineColD.

To develop an integrated database for the global historical tracing and regional real-time monitoring of grapevine cold hardiness/cold damage, we dynamically applied NYUS.2.1 on historical and current temperature records from the weather stations included in GHCNd. The entire process of model generation, data curation, model prediction, and data analysis is briefed in Fig. 1.

### Grapevine cold hardiness prediction model NYUS.2.1

The model used in this study for predicting grapevine cold hardiness, NYUS.2.1, is an updated iteration of the original NYUS.2, an Auto-ML-based model [26]. NYUS.2 was developed using on-site grapevine cold hardiness measurements collected from nine regions across North America between 2003 and 2022 (*n* = 10 157), coupled with features derived from daily temperature data (Fig. 1a). The model was trained using the AutoGluon Auto-ML engine (version 0.7.0) [27]. NYUS.2.1 builds on this foundation by incorporating the original training data along with additional cold hardiness measurements from three locations in New York State during the 2022–2023 and 2023–2024 dormant seasons (*n* = 11277). All cold hardiness data used to develop NYUS.2 and NYUS.2.1 were generated by differential thermal analysis (DTA), a method that detects the temperature that results in the production of a low-temperature exotherm (LTE) that occurs when free water within the primary bud of a grapevine freezes [28, 29]. The average of LTE, termed LT50, representing the temperature that kills 50% of the primary buds, was used to phenotype grapevine cold hardiness [28]. The corresponding weather data for field-collected dormant grapevines were obtained either from the nearest publicly available regional weather stations or from on-site stations deployed directly in the vineyards where cold hardiness measurements were conducted. Detailed information about these weather stations is provided in a previous publication [26]. The training, testing, and feature importance analysis were performed through the same pipeline of NYUS.2 by an upgraded version of AutoGluon (version 1.1.0) [26]. Model performance was evaluated using root mean square error (RMSE) by comparing predicted cold hardiness with observed cold hardiness. The NYUS.2.1 model offers improved predictive capacity for an expanded range of cultivars, increasing from 45 to 54. The information about these cultivars is included in Supplementary Note S1. The prediction relies on the one-hot encoded cultivar information and features extracted from daily maximum and minimum temperatures only.

### Curation of global and regional temperature data

Two types of data curation were conducted to support grapevine cold hardiness predictions, each serving a different purpose: global data curation for historical tracing and regional data curation for real-time monitoring. The data curation process is summarized in Fig. 1b.

To trace historical grapevine cold hardiness, temperature data from weather stations were curated using the GHCNd (https://www.ncei.noaa.gov/products/land-based-station/global-historical-climatology-network-daily). To ensure accurate and efficient predictions while optimizing computational resources, a four-step filtering process was applied to the GHCNd dataset: record type filtering, geographic filtering, temporal filtering, and data quality filtering.

1. Record type filtering: Weather stations lacking continuous records of both daily maximum and minimum temperatures were excluded, as the features for NYUS.2.1 predictions are derived from daily temperature data [26]. Additionally, stations with < 2 years of data were filtered out to ensure data stability.
2. Geographic filtering: Since grapevines are predominantly cultivated between latitudes 30° and 50° [8, 30, 31], we filtered out weather stations outside the 30°–55° latitude band. This range covers both current and potential future viticultural areas where cold stress may be a concern, considering the expected migration of grape and wine industries to cooler regions due to climate change [5, 32–34].
3. Temporal filtering: Stations that ceased recording before 1960 were excluded, along with any data before 1 January 1960, due to issues with accuracy and missing data.
4. Data quality filtering: Temperature records from each remaining weather station were segmented by dormant season (1 September to 1 May for the northern hemisphere and 1 March to 1 November for the southern hemisphere). The dormant seasons with <220 days of temperature record were filtered out to ensure data quality for feature extraction.

The monitoring of grapevine cold hardiness focused on the viticultural regions in North America, where cold damage poses a significant concern [6]. Temperature data for the current season was collected daily from onsite measurements at weather stations curated within the GHCNd dataset with a focus on the viticultural regions with cool or cold dormant seasons in North America. Specifically, data was gathered from GHCNd weather stations located within the geographic boundaries of North Dakota (NW), Maine (NE), Virginia (SE), Colorado (SW), and the states of Washington, Oregon, New Mexico, and Texas in the USA, as well as British Columbia, Québec, Ontario, and Nova Scotia in Canada. No filtering was applied to these weather stations or their data. This daily data collection supports the near real-time updating of grapevine cold hardiness prediction. Additionally, temperature data forecast for the current season was collected from open-meteo (https://github.com/open-meteo/open-meteo) for the forecast of grapevine cold hardiness for the upcoming seven days.

### Feature extraction and grapevine cold hardiness prediction

The feature extraction for temperature data in NYUS.2.1 followed the same methodology as in NYUS.2 [26]. Hourly temperature was estimated based on daily maximum and minimum temperature using ‘stack_hourly_temps’ of the R package ‘chillR’ [35]. Features extracted from hourly temperature data were categorized into four groups: daily temperature descriptors (four features), cumulative temperature descriptors (seven features), exponential weighted moving average temperatures (30 features), and reverse exponential weighted moving average temperatures (30 features). Combined with 54 Boolean-type cultivar features and days in season (number of days after 1 September), a total of 126 features were used for NYUS.2.1 predictions. The detail about these features is available in Supplementary Note S2. The computation process for grapevine cold hardiness is outlined in Fig. 1c. For historical grapevine cold hardiness tracing, NYUS.2.1 was distilled for model prediction using ‘distill = True’ option to manage computational resource demands [27]. Predictions were conducted on all the cultivars included in NYUS.2.1: 21 *Vitis vinifera* cultivars and 33 *V. hybrid* cultivars. We plan to establish an annual update for historical grapevine cold hardiness predictions to ensure the database remains current and accurate. For current grapevine cold hardiness prediction, the full model was deployed, with daily predictions based on continuously updated features. These predictions also covered all cultivars in NYUS.2.1. In addition to NYUS.2.1, two previous benchmark models, NYUS.1 and WAUS.2, were also used for the predictions for ‘Cabernet Sauvignon’, ‘Concord’, and ‘Riesling’ [21, 23].

For both purposes, feature extraction and model prediction for NYUS.2.1 were conducted with paralleled computation by distributing the main task to the available threads through ‘parallel’ and AutoGluon built-in parallelization, respectively [27]. The predicted grapevine cold hardiness of a cultivar is expressed as LT50 [28].

### Cold damage estimation

Potential grapevine cold damage was estimated by comparing the predicted cold hardiness with the minimum temperature of the day. The potential (0–100) that cold damage has occurred is estimated using a symmetric sigmoid function, assuming that there is 10% and 90% potential that damage has occurred when minimum temperature is 2°C above or below the predicted cold hardiness [26].

### Data processing and visualization

The processing, analysis, and visualization of predicted grapevine cold hardiness/cold damage data are briefed in Fig. 1d. For global historical grapevine cold hardiness tracing, predictions from NYUS.2.1 and the estimated cold damage data were organized by the weather station. At each curated station, the maximum cold hardiness and maximum cold damage per dormant season were calculated to visualize the overall impact of climate change over the 64 years between 1960 and 2024. Additionally, the data was segmented by individual seasons to visualize daily cultivar-specific cold hardiness dynamics in response to minimum temperature. For regional real-time grapevine cold hardiness monitoring, daily updated predictions were visualized across all curated weather stations. Geospatial analysis and visualization were conducted using the R package ‘sp’ [36].

### VineColD

All the data generated from global historical cold hardiness tracing and regional real-time cold hardiness monitoring are combined as an integrative database named VineColD. VineColD is currently hosted in an R Shiny-based application at https://cornell-tree-fruit-physiology.shinyapps.io/VineColD/[37]. The global historical cold hardiness dataset is divided by weather station and is stored as .RDS in Box Cloud. Data analysis, visualization, and downloading are facilitated through API pull requests using the built-in function in the application based on the ‘boxr’ R package [38]. Real-time cold hardiness monitoring is hosted in a separate R Shiny-based application, which is mirrored in the VineColD application. All applications built for VineColD feature interactive figures created using the ‘plotly’ R package, interactive weather station maps generated with the ‘leaflet’ R package, and interactive tables made using the ‘DT’ R package [39–41].

## Results and discussion

### Performance of NYUS.2.1 in predicting grapevine cold hardiness

**Figure 2.**
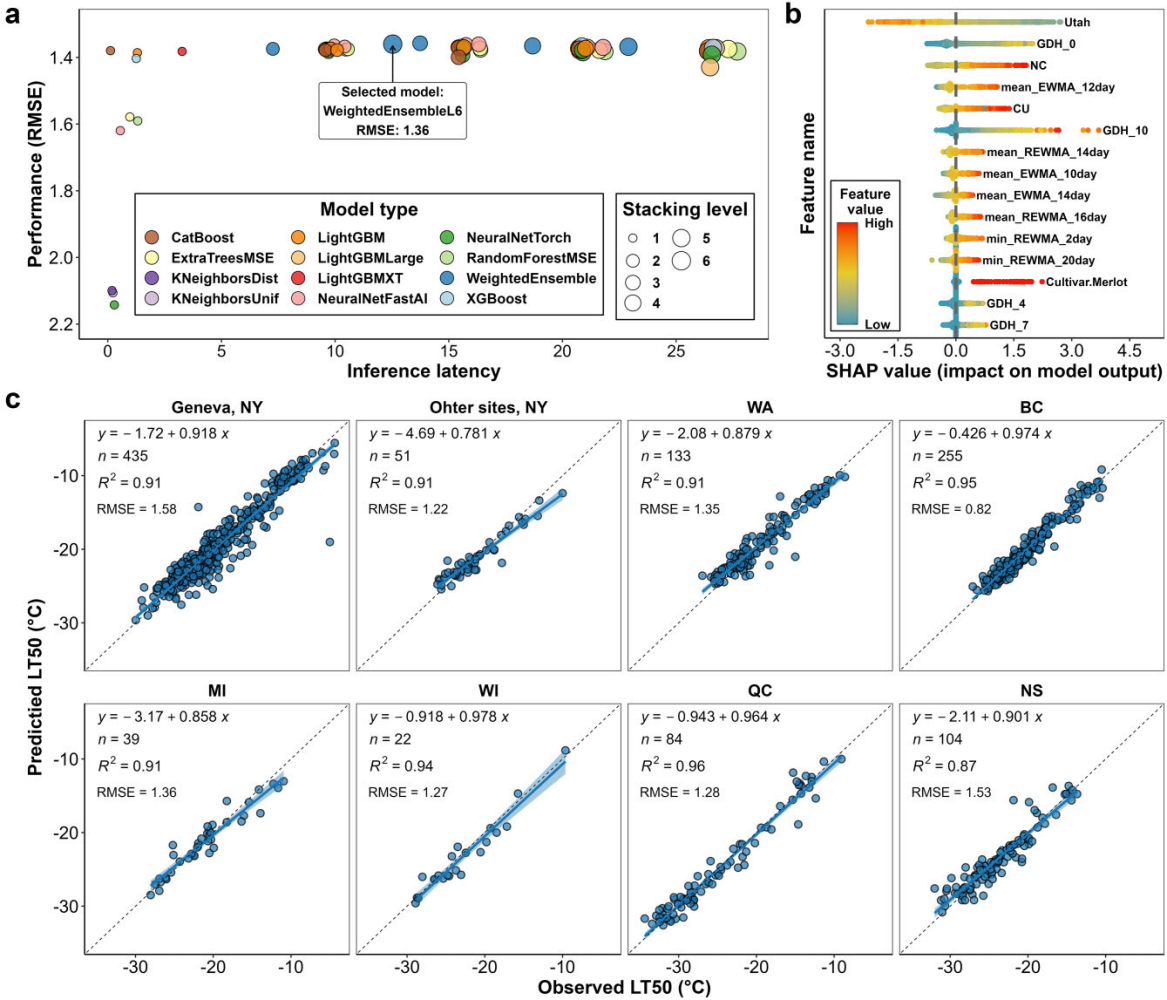
Model selection, feature importance analysis, and model testing for the NYUS.2.1 model. (a) The performance (RMSE) of all models generated during the NYUS.2.1 training process; (b) distribution of SHAP value for the top 15 features in the final model, with feature importance ranked by the mean absolute SHAP values across all testing samples; and (c) performance of NYUS.2.1 in predicting cold hardiness across various subdatasets within the testing data.

During model training, a total of 67 models were generated using various algorithms across different stacking levels (Fig. 2a). The final model for NYUS.2.1, was selected as a ‘WeightedEnsembleL6’, a computationally optimized ensemble model developed after five levels of stacking. This model is composed of eight base or stacker models from stacking levels one and two. This model was chosen for its superior accuracy combined with higher computational efficiency, demonstrated by its relatively low inference latency (Fig. 2a). Feature importance analysis using shapley (SHAP) values [42] revealed that the top 15 most influential features included seven accumulative temperature descriptors: three chilling models (Utah, NC, and CU models) and four growing degree hours (GDH) with base temperatures of 0°C, 4°C, 7°C, and 10°C (Fig. 2b). In model testing with unseen data (10% of the entire dataset, $n = 1127$), NYUS.2.1 demonstrated performance ranging from $RMSE = 0.82^\circ {\mathrm{C}}$ in the ‘BC’ subdataset to $RMSE = 1.58^\circ {\mathrm{C}}$ in the ‘Geneva, NY’ subdataset (Fig. 2c). These prediction errors are minimal as compared to the prediction errors in the utilization/testing of the benchmark models [21, 23–25]. The grapevine cold hardiness predictions by NYUS.2.1 exhibited a balanced distribution around the zero-error slope, which indicates minimal bias towards overestimation or underestimation in these testing regions (Fig. 2c).

### Curation of temperature data

**Figure 3.**
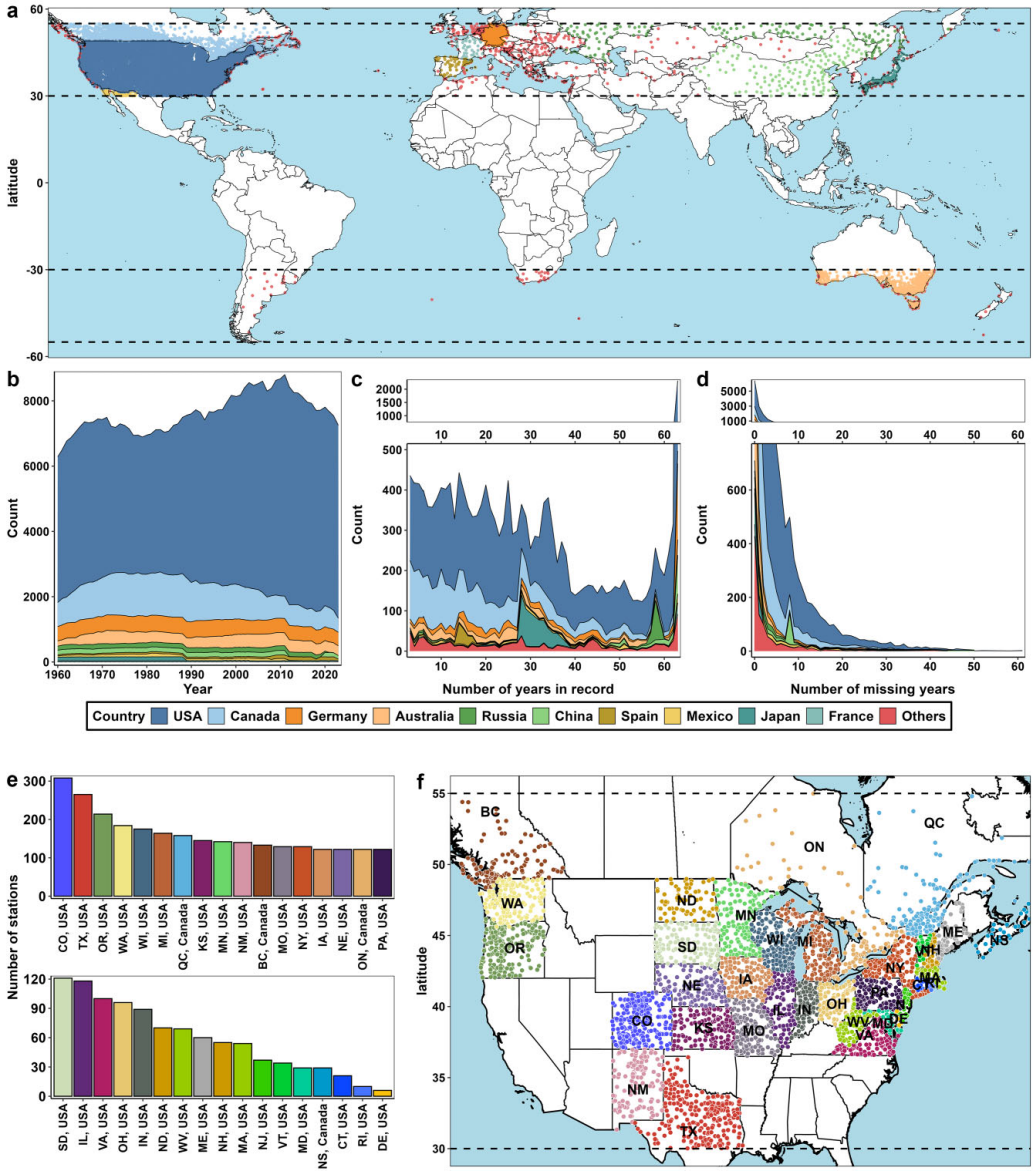
Curation of temperature data for VineColD. (a) Distribution of weather stations in the global data curation process for historical grapevine cold hardiness tracing; (b) number of stations per year; (c) number of years recorded per station; (d) number of missing years per station; (e) number of stations per state/province in the regional data curation for real-time monitoring of grapevine cold hardiness in North America; and (f) distribution of weather stations of the regional curation in North America.

The geographical distribution and completeness of the historical records for the curated weather stations in the VineColD database are detailed in Fig. 3. For global historical grapevine cold hardiness tracing, a total of 17 985 weather stations were curated after the filtering process. The geographical distribution of these stations is shown in Fig. 3a, where each point represents a weather station. These stations are in 63 countries across six continents (Africa, Asia, Europe, North America, South America, and Oceania). Over the 64 years from 1960 to 2024, the number of stations has steadily increased, reaching a peak in the early 2010s before experiencing a decline (Fig. 3b). The detailed breakdown of weather station numbers by country reveals a significant contribution from the USA and Canada (Fig. 3b). The average total record length is 31.8 years, with a notable subset of stations providing data for over six decades, which adds a significant temporal depth to the database (Fig. 3b). The average number of missing years in the records is 4.8 years, and the histogram of missing years shows a scale-free distribution, indicating that most curated stations have maintained comprehensive records with minimal annual gaps (Fig. 3d).

The regional real-time grapevine cold hardiness monitoring system was launched in the early 2022–2023 dormant season and has since provided cold hardiness predictions across three dormant seasons. In the current system, a total of 3772 weather stations have been curated, evenly distributed across the monitored regions (Fig. 3e and f). Colorado (CO), Texas (TX), Oregon (OR), Washington (WA), and Wisconsin (WI) are the top five regions contributing the most weather stations (Fig. 3e).

Overall, the extensive curation of weather stations in VineColD ensures a historically rich and geographically comprehensive dataset, providing a robust foundation for accurate and reliable grapevine cold hardiness modelling and climate impact studies.

### Functions of different components in VineColD

**Figure 4.**
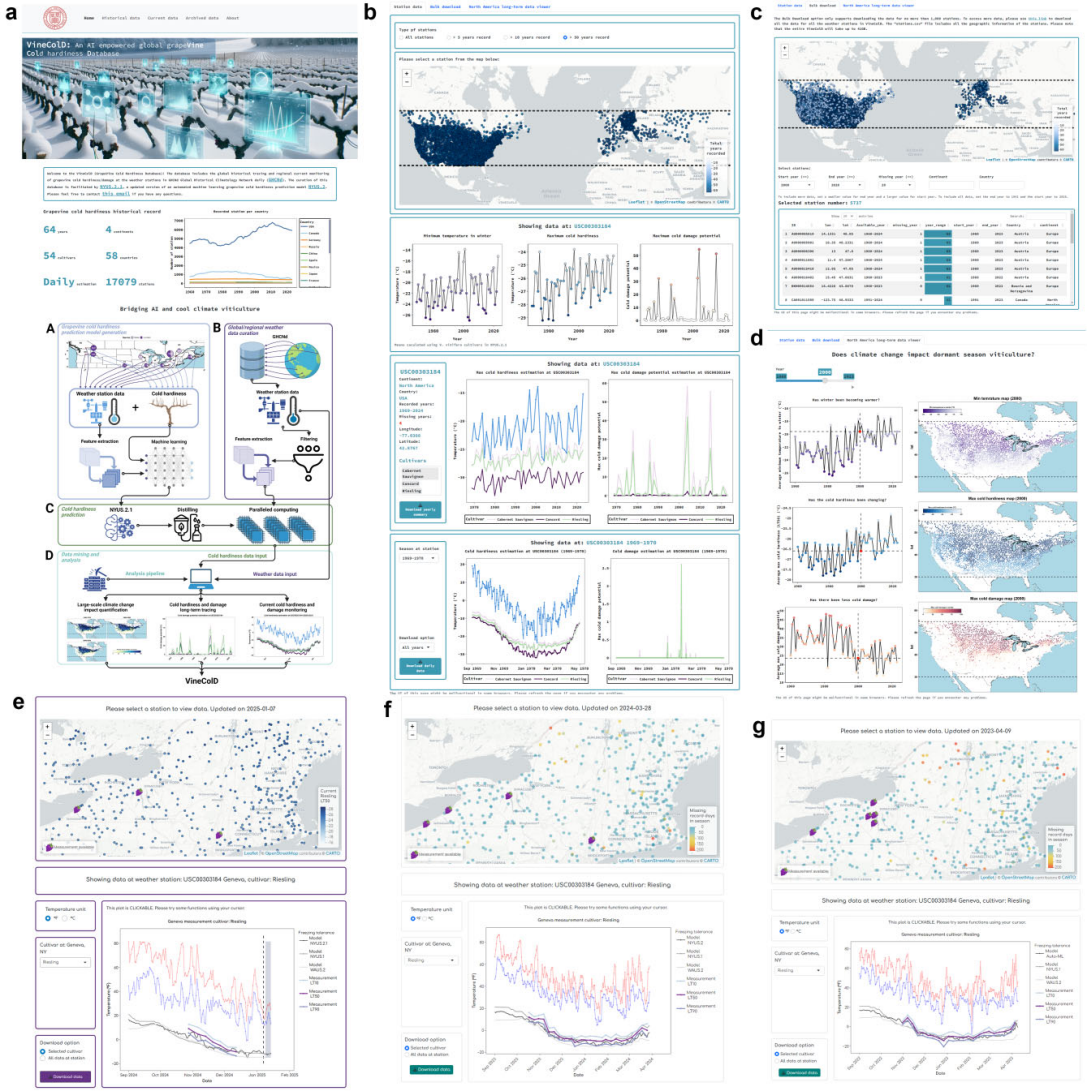
User interface of the R Shiny-based application containing VineColD. (a) Home page of the application; (b) composition of ‘Station data’ under ‘Historical data’ tab; (c) composition of ‘Bulk download’ under ‘Historical data’ tab; (d) composition of ‘North America long-term data viewer’ under ‘Historical data’ tab; (e) current user interface of the regional real-time grapevine cold hardiness monitoring application; (f) archived regional real-time grapevine cold hardiness monitoring application in the 2023–2024 dormant season; and (g) archived regional real-time grapevine cold hardiness monitoring application in the 2022–2023 dormant season.

The predicted cold hardiness, estimated cold damage, historical temperature data, and current temperature data were combined and used as the data input for VineColD. VineColD is deployed in a user interface that provides various data acquisition options and data visualization methods towards the curation and interactive analysis of global historical and regional real-time grapevine cold hardiness (Fig. 4a). The major components of the VineColD application are ‘Historical data’, ‘Current data’, and ‘Archived data’. ‘Historical data’ supports three functions: ‘Station data’ allows users to select individual weather stations on a station map, download selected cold hardiness/damage data, and visualize seasonal maximum cold hardiness, maximum cold damage summaries, and detailed cold hardiness/cold damage dynamics for each season (Fig. 4b); ‘Bulk download’ enables users to download cold hardiness data from grouped weather stations based on various filtering criteria such as start year, end year, missing year, continent, and country (Fig. 4c); ‘North America long-term data viewer’ provides visualizations of average minimum temperatures, average maximum predicted cold hardiness, and average maximum estimated cold damage for all curated weather stations in North America between 1960 and 2024, along with corresponding station maps showing their geographic distribution (Fig. 4d). ‘Current data’ hosts a daily-updated application for the current grapevine cold hardiness with predicted cold hardiness from three models, NYUS.2.1, NYUS.1, and WAUS.2, along with uploaded on-site measurements in the viticultural regions with cool or cold dormant seasons in the USA and Canada (Fig. 4e). The system automatically begins updating each year on 15 September. The application enables near real-time prediction of grapevine cold hardiness and includes a ‘Data Upload’ portal where researchers can contribute on-site cold hardiness measurements. These data are displayed in the application within 24 h and are downloadable, which provides ground-truth cold hardiness measurement along with model predictions. At the end of each dormant season, the uploaded data will be processed using the same feature extraction method as the training data and integrated into the training dataset for annual model updates. In the 2024–2025 dormant season, five North American research groups contributed over 800 LT50 data points through this system. ‘Archived data’ hosts old versions of the ‘Current data’ application from the 2022–2023 and the 2023–2024 dormant seasons, including all the predicted and uploaded measurement data (Fig. 4f and g).

### Case study: using VineColD data to quantify the impact of climate change on grapevine cold damage in North America (1960–2024)

**Figure 5.**
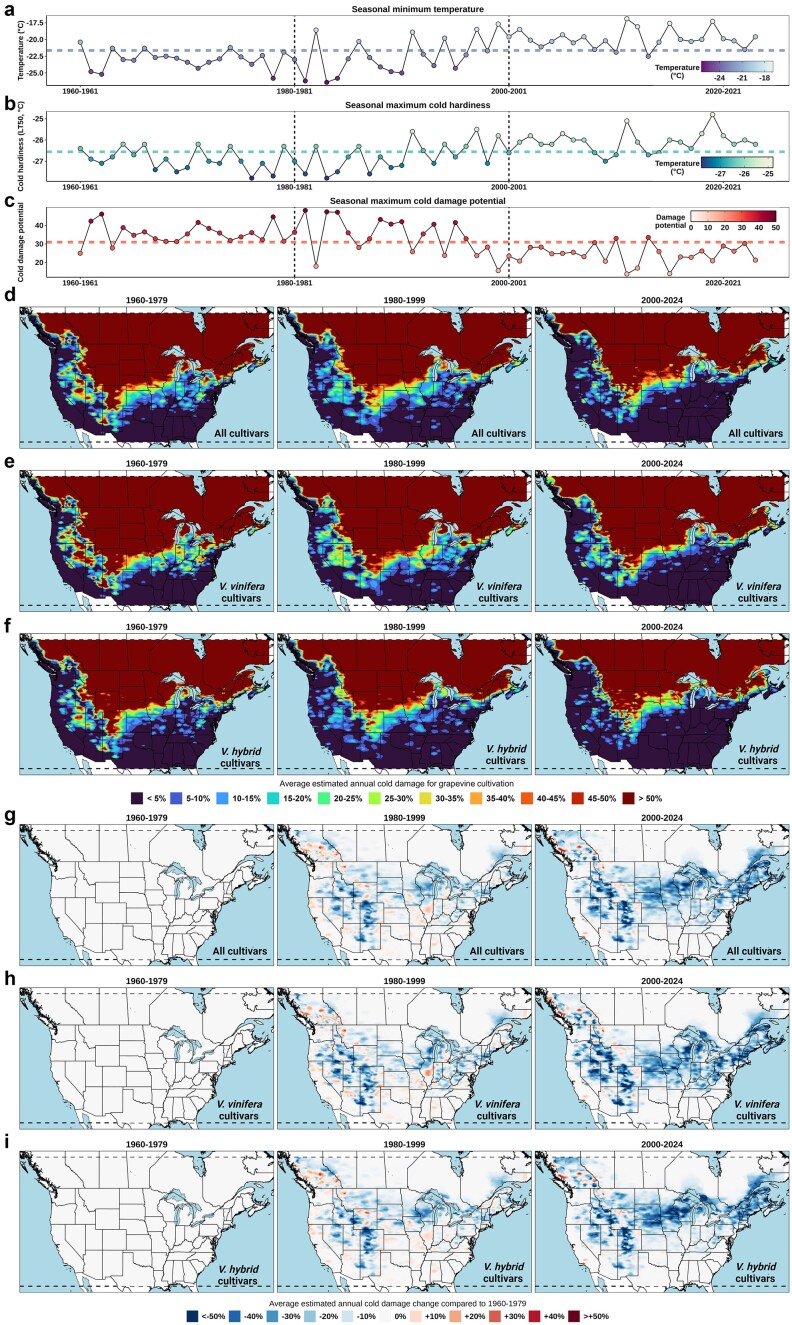
Quantification of the impact of climate change on grapevine cold damage in North America from 1960 to 2024. (a) Average seasonally minimum temperature across all the curated weather stations. The line in the middle represents the average of the 64 dormant seasons; (b) average seasonally maximum estimated cold hardiness of all cultivars in NYUS.2.1 at all curated weather stations; (c) average seasonal maximum estimated cold damage potential of all cultivars in NYUS.2.1 at all curated weather stations; (d) gridded estimation of averaged seasonal cold damage of all cultivars included in NYUS.2.1 in North America, segmented into three time windows: 1960–1979, 1980–1999, and 2000–2024; (e) gridded estimation of averaged seasonal cold damage of *V. vinifera* cultivars included in NYUS.2.1 in North America between 1960 and 2024; (f) gridded estimation of averaged seasonal cold damage of *V. hybrid* cultivars included in NYUS.2.1 in North America between 1960 and 2024; (g) gridded estimation of averaged annual cold damage change of all cultivars compared to 1960–1979; (h) gridded estimation of averaged annual cold damage change of *V. vinifera* cultivars compared to 1960–1979; and (i) gridded estimation of averaged annual cold damage change of *V. hybrid* compared to 1960–1979.

In our first attempt to utilize VineColD data, we conducted a case study analyzing grapevine cold hardiness and damage across North America using data from curated weather stations between 1960 and 2024. The goal was to identify trends and to compare grapevine cold hardiness and damage between different time periods to quantify the impact of climate change on cold damage during the dormant season. To facilitate this comparison, the period of 1960–2024 was segmented into three different time windows: 1960–1979, 1980–1999, and 2000–2024. For each window and at each curated weather station, we calculated the average minimum temperature, average maximum cold hardiness, and average maximum cold damage during the dormant season. These calculations were performed for all cultivars, as well as specifically for *V. vinifera* and *V. hybrid* cultivars included in NYUS.2.1, to approximate the low-temperature boundaries for different grapevine species under cold stress. To enhance the spatial resolution of the analysis, we implemented a k-nearest neighbors (KNN) model using cold damage data from each window, with only longitude and latitude as input features [43]. This approach allowed us to extrapolate site-specific observations to a gridded spatial scale, resulting in the high-resolution maps presented in Fig. 5.

The regional analysis of estimated cold damage revealed significant shifts across North America from 1960 to 2024 (Fig. 5). During the initial period of 1960–1979, average seasonal minimum temperatures at curated weather stations remained consistently low, ranging from −25.5°C to −20.4°C (Fig. 5a). Despite superior average seasonal maximum cold hardiness during this time, the average seasonal maximum cold damage potential was also high (Fig. 5b and c). As a result, grapevine cultivation faced substantial cold damage risk, particularly in the upper Midwest, Great Lakes, and Northeast regions, with localized pockets of higher risk in the Rocky Mountain areas for *V. vinifera* (Fig. 5e). For *V. hybrid* cultivars, the cold damage risk was more evenly distributed across the upper Midwest and Northeast, reflecting their broader cultivation potential and different cold hardiness characteristics (Fig. 5f).

In the 1980–1999 window, average seasonal minimum temperatures began to exhibit an ‘unstable and increasing’ trend, characterized by considerable fluctuations between years (Fig. 5a). Similar fluctuating trends were observed in average seasonal maximum cold hardiness and cold damage potential (Fig. 5b and c). Consequently, some small high-risk pockets emerged during this period in regions such as the lower Midwest and South regions of the USA and British Columbia Province in Canada (Fig. 5d to i). A regional decrease in cold damage risk was also observed in other regions such as the upper Midwest, Great Lakes, and Northeast regions of the USA, as well as the lower regions of Ontario Province in Canada (Fig. 5d–i). From 2000 to 2024, the trend of decreasing cold damage became more prevalent (Fig. 5c–i). For *V. vinifera* cultivation, this decrease was evident in the Pacific Northwest, parts of the Midwest, and the Northeast, where cold damage risk fell <20% in areas that previously faced higher risks (Fig. 5e and h). Similarly, *V. hybrid* cultivars showed a notable decrease in cold damage risk in the Appalachian Highlands and coastal areas of the Pacific Northwest (Fig. 5f and i). Notably, some coastal areas in the Maritime provinces of Canada and the New England states in the USA indicated potential for *V. hybrid* cultivation during this period (Fig. 5f and i). Assuming regions with <20% annual cold damage are suitable for grapevine cultivation, our simulation predicts a 28.8% and 17.2% increase in the feasible land area in the studied region for cultivating *V. vinifera* and *V. hybrid* cultivars, respectively, when comparing the 2000–2024 window to the 1960–1979 window.

#### Future potential and challenges

The NYUS.2.1 model and the VineColD platform represent a novel data-driven approach to studying viticulture and supporting the global grape and wine industry. NYUS.2.1 is currently the most up-to-date grapevine cold hardiness model developed through the integration of extensive field-based cold hardiness data, Auto-ML, and advanced weather feature extraction. To our knowledge, it is the first and only Auto-ML-based cold hardiness model for a perennial fruit crop that has been both widely deployed and validated across diverse geographic locations. Given that the physiological mechanism underlying cold acclimation and deacclimation are broadly conserved among temperate woody perennials [15], we believe the cold hardiness dynamics captured by NYUS.2.1 may be transferable to other species if appropriate cold hardiness data are available. Beyond cold hardiness, the modelling framework, including weather feature extraction and Auto-ML, can be applied to a wide range of physiological traits to better understand plant-environment interactions or to deliver practical, data-informed tools for stakeholders. Initial efforts to expand this framework are underway. As some preliminary tests, we have adapted NYUS.2.1 to model bud cold hardiness in apple (https://github.com/imbaterry11/NYUS.2.1_apple_grape), and the same modelling pipeline has been used to predict hybrid maize yield across multiple environments (https://github.com/imbaterry11/UFEED_Maize_GxE).

While the development of NYUS.2.1 and VineColD is promising, we also recognize some limitations of the model and the database. As a measurement-based machine learning model, NYUS.2.1 relies entirely on cold hardiness data from DTA measurements and local weather data. One major limitation of DTA is the difficulty in distinguishing LTE from high-temperature exotherms (HTE), especially during the rapid deacclimation period near budbreak. HTEs typically occur between −6°C and −10°C, which could overlap with the higher LTE (less negative) in this period, introducing uncertainty into late-season measurements. Thus, our dataset contains relatively few high-quality measurements during the late dormant season, potentially impairing the model’s accuracy during that period. As a result, NYUS.2.1 and its predecessor NYUS.2 perform best during early to mid-winter but may tend to overestimate cold hardiness in late winter and early spring [26]. Furthermore, all cold hardiness data used to develop the model are from North American viticultural regions with cool to cold dormant seasons. To date, no comparable LT50 datasets are available from other continents such as Europe, South America, or Australia. As a result, the model performance outside North America, particularly in regions with mild winters, remains untested and may carry greater uncertainty. While grapevines in these warmer climates likely still develop cold hardiness, the lack of winter cold injury has historically limited the need for measurement, and thus model calibration and validation in these regions is lacking. We openly acknowledge this limitation within the VineColD database and its associated applications. However, by serving as an open data-sharing platform, VineColD encourages global contributions of cold hardiness data, especially from late-season measurements and underrepresented climate regions. These future efforts will be crucial for improving the model's generalizability and extending its utility to a broader range of viticultural regions.

## Conclusion

This study presents VineColD, a novel database that integrates historical and real-time data to evaluate grapevine cold hardiness, representing a significant advancement in dormant season grapevine physiology modelling and offering a practical application for both researchers and industry stakeholders. To our knowledge, the current version of VineColD represents the first and the only large-scale grapevine cold hardiness database in the world. VineColD data estimates for the quantification of climate change’s impact on grapevine cold damage in North America indicate that there has been an increase in the land area feasible for grapevine cultivation if only considering cold damage as the limiting factor. Looking forward, the expansion VineColD, coupled with the annual update of NYUS.2.1 to cover more cultivars and viticultural areas, could provide a more comprehensive database for global grapevine cold hardiness/cold damage. VineColD sets a foundation for future research and development in viticulture, offering a robust platform for studying and mitigating the effects of climate change on grapevine cultivation. This innovative approach could help develop more resilient agricultural practices and contribute to the sustainability and growth of the viticulture industry worldwide.

## Supplementary Material

baaf055_Supplemental_File

## Data Availability

VineColD is publicly available at https://cornell-tree-fruit-physiology.shinyapps.io/VineColD/. The current regional real-time grapevine cold hardiness monitoring system is publicly available at https://cornell-tree-fruit-physiology.shinyapps.io/North_America_Grape_Freezing_Tolerance/. The model training, model testing, feature importance analysis, and model prediction are publicly available at https://github.com/imbaterry11/NYUS.2.
