# Supplementary material for: VineColD: an integrative database for global historical tracing and real-time monitoring of grapevine cold hardiness
Source: Database (Oxford). 2025 Sep 27;2025:baaf055. doi: 10.1093/database/baaf055 (PMC12475903; doi:10.1093/database/baaf055)
Supplement: baaf055_Supplemental_File [file baaf055_supplemental_file.docx]

Table 1. Summary of PES design literature and its overlap with our study (by May 2025), sorted by their overlap with common (Sections 2.1-2.4) and novel (Sections 3 and 4) themes discussed in the manuscript.

|  | **Section 2.1** | **Section 2.2** | **Section 2.3** | **Section 2.4** | **Section 3** | **Section 4** |  |
| --- | --- | --- | --- | --- | --- | --- | --- |
| **Authors** | Conditionality - Choice of proxy | Impact on social motivation | Contract arrangements | Spatial targeting | Future PES cost estimates | Climate change impacts on PES design | **Country/Geographic region** |
| Lliso et al | 0 | 1 | 0 | 0 | 0 | 0 | Colombia |
| Abildtrup et al | 1 | 1 | 0 | 0 | 0 | 0 | France |
| Adhikari and Boag | 1 | 0 | 1 | 0 | 0 | 0 | Global |
| Chobotova | 0 | 1 | 1 | 0 | 0 | 0 | Central and Eastern EU |
| Brownson et al | 1 | 0 | 1 | 0 | 0 | 0 | Costa Rica |
| Baylis et al | 1 | 0 | 1 | 0 | 0 | 0 | US; EU |
| Wuepper et al | 1 | 0 | 1 | 0 | 0 | 0 | Switzerland |
| Pagiola et al | 1 | 0 | 1 | 0 | 0 | 0 | Colombia |
| Pagiola et al | 1 | 0 | 1 | 0 | 0 | 0 | Nicaragua |
| Jayachandran | 1 | 0 | 1 | 0 | 0 | 0 | Uganda |
| Peskett et al | 0 | 1 | 1 | 0 | 0 | 0 | Uganda |
| Rode et al | 0 | 1 | 0 | 1 | 0 | 0 | Global |
| Daniels et al | 1 | 0 | 0 | 1 | 0 | 0 | Costa Rica |
| Kroeger | 1 | 0 | 0 | 1 | 0 | 0 | Global |
| Lambin et al | 0 | 1 | 0 | 1 | 0 | 0 | Global |
| Muradian et al | 0 | 1 | 0 | 1 | 0 | 0 | South America; Global |
| Pagiola | 1 | 0 | 0 | 1 | 0 | 0 | Latin America |
| Schomers and Matzdorf | 1 | 0 | 0 | 1 | 0 | 0 | Global |
| Zabel and Roe | 1 | 0 | 0 | 0 | 0 | 0 | Global |
| Corbera et al | 1 | 0 | 1 | 1 | 0 | 0 | Mexico |
| de Fries and Hanley | 0 | 0 | 1 | 1 | 0 | 0 | Global |
| Engel et al | 1 | 0 | 1 | 1 | 0 | 0 | Global |
| Ferraro | 1 | 0 | 1 | 1 | 0 | 0 | Global |
| Jayachandran et al | 0 | 1 | 1 | 1 | 0 | 0 | Uganda |
| Jost and Gentes | 0 | 1 | 1 | 1 | 0 | 0 | South and Latin America |
| Leimona et al | 1 | 1 | 1 | 0 | 0 | 0 | Indonesia |
| Mahanty et al | 1 | 0 | 1 | 1 | 0 | 0 | Global |
| Matzdorf and Lorenz | 1 | 1 | 1 | 0 | 0 | 0 | Germany |
| Nelson et al | 1 | 0 | 1 | 1 | 0 | 0 | Tanzania |
| Pattanayak et al | 1 | 1 | 1 | 0 | 0 | 0 | Global |
| Primmer et al | 0 | 0 | 1 | 1 | 0 | 0 | Finland |
| Sierra and Russman | 1 | 0 | 1 | 1 | 0 | 0 | Costa Rica |
| Thompson | 1 | 0 | 1 | 1 | 0 | 0 | Global |
| Vorlaufer et al | 1 | 1 | 1 | 0 | 0 | 0 | Uganda |
| Windle and Rolfe | 1 | 0 | 1 | 0 | 0 | 0 | Australia |
| Burton et al | 1 | 1 | 1 | 0 | 0 | 0 | EU; UK |
| Erbaugh | 1 | 1 | 1 | 1 | 0 | 0 | Indonesia |
| Hayes et al | 1 | 1 | 1 | 0 | 0 | 0 | Ecuador |
| Izquierdo-Tort et al | 1 | 1 | 1 | 1 | 0 | 0 | Mexico |
| Jack et al | 1 | 1 | 1 | 0 | 0 | 0 | Global; US |
| Klimek et al | 1 | 0 | 1 | 1 | 0 | 0 | EU |
| Montoya-Zumaeta et al | 1 | 0 | 1 | 1 | 0 | 0 | Peru |
| Persson and Alpizar | 1 | 1 | 1 | 0 | 0 | 0 | Global |
| Pindilli and Casey | 1 | 0 | 1 | 1 | 0 | 0 | US |
| Sommerville et al | 1 | 1 | 1 | 1 | 0 | 0 | Madagascar |
| Šumrada et al | 1 | 1 | 1 | 1 | 0 | 0 | Slovenia |
| Wunder et al | 1 | 1 | 1 | 1 | 0 | 0 | Global |
| Wunder et al | 1 | 1 | 1 | 1 | 0 | 0 | Global |
| Tennent and Lockie | 0 | 1 | 1 | 1 | 0 | 0 | Australia |
| Salzman et al | 1 | 0 | 0 | 0 | 1 | 0 | Global |
| Gauvin et al | 1 | 0 | 0 | 1 | 1 | 0 | China |
| Turpie et al | 1 | 0 | 1 | 0 | 1 | 0 | South Africa |
| Aza et al | 1 | 1 | 1 | 0 | 1 | 0 | Brazil |
| Claassen et al | 1 | 0 | 1 | 0 | 1 | 0 | US |
| Alix-Garcia et al | 1 | 1 | 1 | 1 | 1 | 0 | Mexico |
| Börner et al | 1 | 1 | 1 | 1 | 0 | 0 | Global |
| Clements et al | 1 | 1 | 1 | 1 | 1 | 0 | Cambodia |
| Hanley et al | 1 | 0 | 1 | 1 | 1 | 0 | UK |
| Hellerstein | 1 | 0 | 1 | 1 | 1 | 0 | US |
| Palm-Forster et al | 1 | 0 | 1 | 1 | 1 | 0 | US |
| Wünscher et al | 1 | 0 | 1 | 1 | 1 | 0 | Costa Rica |
| Kangas and Ollikainen | 1 | 0 | 0 | 1 | 1 | 1 | Finland |

Table 2. Data sources, brief description of analysis and the corresponding sections

| Data | Description of data | Source |
| --- | --- | --- |
| METSO basic statistics | Total area protected under METSO, summarized by year and contract type showing area protected (ha) and cost of contract per hectare (**fig 2**) | Natural Resource Institute Finland  [www.statdb.luke.fi](https://statdb.luke.fi/PxWeb/pxweb/en/LUKE/LUKE__04%20Metsa__02%20Rakenne%20ja%20tuotanto__04%20Metsien%20suojelu/11_Etela_suomen_metso_ely.px/?rxid=001bc7da-70f4-47c4-a6c2-c9100d8b50db) |
| Timber volume | Mean timber volume of forest stands, collated from multiple sources using satellite images, airborne laser scanning and field surveys, and aggregated into 96 x 96 m raster pixels. (**fig 3**) | Virkkala et al. 2022 |
| Conservation priority | Map of unprotected forested areas in Finland, ranked from highest to lowest priority for conservation. The prioritization was done by Forsius et al*.* 2023 using a spatial conservation optimization software Zonation 5.1 (Moilanen et al. 2022). (**fig 3** and **table 2)** | Forsius et al. 2023 |
| Spatial data on public lands | Privately owned layer created by inverting from all publicly owned and protected areas in Finland. The proportion of priority conservation areas calculated for national and regional protection targets of 10/30 that fall on public and private lands. (**table 2)** | Metsähallitus (Finnish Parks and Wildlife) |
| Predicted timber volume | Underlying data comes from results of the predictive models by Triviño et al. 2023a under two different climate scenarios. (**fig 4)** | Triviño et al. 2023a |
